# The Hedgehog Receptor Patched Is Involved in Cholesterol Transport

**DOI:** 10.1371/journal.pone.0023834

**Published:** 2011-09-08

**Authors:** Michel Bidet, Olivier Joubert, Benoit Lacombe, Marine Ciantar, Rony Nehmé, Patrick Mollat, Lionel Brétillon, Hélène Faure, Robert Bittman, Martial Ruat, Isabelle Mus-Veteau

**Affiliations:** 1 Université de Nice - Sophia Antipolis, CNRS-UMR 6543, Institute of Developmental Biology and Cancer, Nice, France; 2 Université Henri Poincaré- Nancy 1, EA 3452, Faculté de Pharmacie, Nancy, France; 3 Laboratory of Molecular Biology, Medical Research Council (MRC), Cambridge, United Kingdom; 4 Galapagos SASU, Romainville, France; 5 Université de Bourgogne, CNRS-INRA, Centre des Sciences du Goût et de l'Alimentation, Dijon, France; 6 CNRS, UPR-3294, Laboratoire de Neurobiologie et Développement, Institut de Neurobiologie Alfred Fessard IFR2118, Gif-sur-Yvette, France; 7 Department of Chemistry and Biochemistry, Queens College of the City University of New York, Flushing, New York, United States of America; Institut Curie, France

## Abstract

**Background:**

Sonic hedgehog (Shh) signaling plays a crucial role in growth and patterning during embryonic development, and also in stem cell maintenance and tissue regeneration in adults. Aberrant Shh pathway activation is involved in the development of many tumors, and one of the most affected Shh signaling steps found in these tumors is the regulation of the signaling receptor Smoothened by the Shh receptor Patched. In the present work, we investigated Patched activity and the mechanism by which Patched inhibits Smoothened.

**Methodology/Principal Findings:**

Using the well-known Shh-responding cell line of mouse fibroblasts NIH 3T3, we first observed that enhancement of the intracellular cholesterol concentration induces Smoothened enrichment in the plasma membrane, which is a crucial step for the signaling activation. We found that binding of Shh protein to its receptor Patched, which involves Patched internalization, increases the intracellular concentration of cholesterol and decreases the efflux of a fluorescent cholesterol derivative (BODIPY-cholesterol) from these cells. Treatment of fibroblasts with cyclopamine, an antagonist of Shh signaling, inhibits Patched expression and reduces BODIPY-cholesterol efflux, while treatment with the Shh pathway agonist SAG enhances Patched protein expression and BODIPY-cholesterol efflux. We also show that over-expression of human Patched in the yeast *S. cerevisiae* results in a significant boost of BODIPY-cholesterol efflux. Furthermore, we demonstrate that purified Patched binds to cholesterol, and that the interaction of Shh with Patched inhibits the binding of Patched to cholesterol.

**Conclusion/Significance:**

Our results suggest that Patched may contribute to cholesterol efflux from cells, and to modulation of the intracellular cholesterol concentration. This activity is likely responsible for the inhibition of the enrichment of Smoothened in the plasma membrane, which is an important step in Shh pathway activation.

## Introduction

The Hedgehog (Hh) pathway plays a crucial role in growth and patterning during embryonic development. Consequently, defects in Hh signaling involve human congenital malformations and disorders such as holoprosencephaly (HPE) and Gorlin's syndrome [1], [2], [3], [4]. In adults, recent studies suggest a role of this pathway in stem cell self-renewal and in the mobilization of endogenous stem cells for tissue repair and regeneration following injury and disease [5], [6], [7]. Mutations in the Hh pathway components have been identified in basal cell carcinoma, medulloblastoma, and rhabdomyosarcoma, and aberrant activity of the pathway has been shown to be involved in the development of many other tumors (lung, esophagus, stomach, pancreas, biliary tract, breast, prostate, and brain) [8]. Many of these tumors contain cancer stem cells which retain self-renewal properties, representing a never-ending reservoir for the maintenance of the tumor mass [8].

In Hh-secreting cells, newly made Hh protein undergoes auto-processing and lipid modification, resulting in the secretion of a fully active N-terminal Hh signaling domain (HhN) of 19 kDa modified by a palmitoyl group and a cholesterol molecule, respectively, in its N- and C-termini [9]. Secreted HhN is able to act over a long range by a mechanism that is not yet completely understood. The different HhN proteins (ShhN, IhhN, and DhhN) are secreted at various stages of development, and initiate signaling in receiving cells by binding to the Hh-receptor Patched (Ptc). In vertebrates, the interaction between HhN and Ptc relieves the inhibition of the signal transducer Smoothened (Smo), which is then re-localized and enriched at the plasma membrane and activated. This triggers a cascade of downstream events that culminates in the activation or derepression of target gene transcription through the zinc finger transcription factors glioma-associated oncogenes (Gli) [1]. In the absence of secreted Hh, the pathway is turned off due to the inhibition exerted by Ptc on Smo. Ptc, Smo, Su(Fu), and Gli have been detected in primary cilia, which are present in nearly all vertebrate cells to transmit information from the environment [10], [11]. Several components of the transport machinery required for the assembly and maintenance of cilia were reported to be essential for Hh signaling [12], [13], [14].

The regulation of Smo activation by Ptc appears to be one of the most disrupted steps in the Hh pathway related to human cancers, and the mechanism by which Ptc represses Smo remains unresolved. Taipale and co-workers showed in 2002 that Ptc inhibits Smo sub-stoichiometrically, thereby avoiding direct interaction between Ptc and Smo [15]. Several small molecules modulate Hh signaling through direct binding to Smo, and some Smo antagonists are in clinical trials for treating tumors [8], [16]. Moreover, Smo would be repressed by molecules such as vitamin D_3_ [17], and activated by oxysterols [13], [18], [19] and phosphatidylinositol 4-phosphate [20]. On the basis of these observations and the sequence homology of Ptc with bacterial transmembrane transporters, Ptc has been proposed to function as a pump that would change the concentration of a small molecule involved in Smo activation or inhibition [15], [21]. However, the transport activity of Ptc has not yet been demonstrated, the identity of the small molecule responsible for the physiological Ptc/Smo regulation is still unknown, and the mechanism by which Ptc regulates Smo activation remains to be elucidated.

In the present study, we report that Ptc is likely involved in the efflux of cholesterol from cells, and we propose that this activity may contribute to Smo repression.

## Materials and Methods

### Cell cultures

NIH 3T3 mouse fibroblast cells (ATCC CRL-1658) were grown in DMEM medium (Invitrogen) supplemented with 10% fetal bovine serum, 100 U/mL penicillin, and 100 µg/mL streptomycin, at 37°C in a 5% CO_2_/95% air water-saturated atmosphere. Depending on the experiment, 30 nM ShhN, 10 µM purmorphamine (Alexis Biochemicals), 10 µM cyclopamine (Jentaur), or 10 µM cholesterol (Sigma) were added to the culture medium when the cells were 80% confluent.

K699 *S. cerevisiae* yeast strain (Mata, ura3, and leu 2–3) transformed with YEpPMAhPtc-MAP, YEpPMAhSmo-MAP, or YEpPMAmMyo-MAP expression vector were grown as described [22] at 18°C until OD_600_ = 5–7.

### RNA isolation, reverse transcription and quantitative RT-PCR (qRT-PCR)

Total cellular RNA was extracted from NIH 3T3 cells using the TRIzol reagent according to the manufacturer's instructions (Invitrogen). One µg of total RNA from control or treated samples were reverse-transcripted with 50 nmol of oligo (dT) using M-MLV reverse transcriptase (RT) (Invitrogen) following the manufacturer's protocol. Real-time quantitative RT-PCR analyses were performed with a Stratagene Mx3000p system and Mesa Green qPCR MasterMix Plus for SYBR® (RT-SY2X-03+WOULR, Eurogentec). Briefly, 100 ng of reverse-transcripted RNA from each sample was mixed with appropriate concentrations of tested gene primers and the Mesa Green qPCR MasterMix. Primers were designed using Primer3 input *patched1* sense AGCTGTGGGTGGAAGTTGGT, antisense TCCGTGATAAGTTCCCCTGA, 18S mRNA sense CGCCGCTAGACGTAGAATTCT, antisense CATTCTTGGCAAATGCTTTGC. PCR amplifications were carried out as follows: 10 min at 95°C; 45 cycles (30 s at 95°C, 60 s at 60°C, and 60 s at 72°C). For each condition, expression was quantified in duplicate, and 18S mRNA was used as the endogenous control in the comparative cycle threshold (C_T_) method. Data were expressed as relative expression ratio.

### NIH 3T3 membrane preparation

All steps were performed at 4°C. NIH 3T3 cells were grown on 100-mm Petri dishes to confluence, collected, centrifuged, washed two times with PBS and one time with water, and re-suspended in hypotonic buffer containing 50 mM Tris-HCl, pH 7.5, 1 mM EDTA, and proteases inhibitor cocktail (PIC, Roche). After 10 min on ice, the cells were broken by passages through a syringe. Cellular remains were pelleted for 10 min at 430 *g* and the supernatant was centrifuged for 30 min at 20,000 *g* to collect heavy membranes, essentially plasma membranes.

### NIH 3T3 total extract preparation

Cells were collected, pulled down at maximum speed for a few seconds, washed one time with PBS, and then re-suspended in RIPA buffer (50 mM Tris-HCl, pH 7.4, 150 mM NaCl, 1 mM PMSF, 1 mM EDTA, 1% Triton, 1% sodium deoxycholate, 0.1% SDS, PIC). Unsolubilized cells and cellular remains were pelleted, and the supernatants were collected.

### Protein quantification

Protein concentrations were determined by the Bradford method using a Bio-Rad kit.

### Purification of ShhN protein

Mouse fibroblast L cells (LTK-P2 cells obtained from RIKEN Cell Bank (RCB0208), Japan) stably expressing the active amino-terminal domain of the murine Shh protein corresponding to amino acids 25 to 198 (ShhN) were grown in suspension, and ShhN protein was purified from 5 L of conditioned culture medium as described previously [22]. The ability of purified ShhN to activate the Hh pathway was assessed on C3H10T1/2 cells (from ATCC) by alkaline phosphatase activity and Gli responsive element activity as described previously [22].

### Purification of hPtc and mMyo

Yeasts expressing human Ptc (hPtc) or mouse Myodulin (mMyo) were cultured until an OD_600_ of 7 was reached. Membranes were prepared and solubilized with 20 mM dodecyl-β-D-maltoside (DDM, Calbiochem). hPtc and mMyo were purified on a calmodulin-Sepharose resin (Amersham) as described previously [22], [23], [24].

### Generation of polyclonal antiserum to the mouse Patched

A DNA encoding part of the carboxyl-terminal domain of the mouse Patched (residues 1321–1427) was amplified by PCR and fused to GST in pGEX-4T-1. The fusion protein was purified and antiserum was produced in a rabbit as previously described [25]. The antiserum (Ab130) has been tested on HEK293 cells (from ATCC CRL-1573) transiently transfected with mouse Ptc cDNA and was shown to be highly specific for Ptc (Fig. S1).

### SDS-PAGE and Western blotting

Protein samples were separated on 8% SDS-PAGE and transferred to nitrocellulose membranes (Amersham) using standard techniques. After 1 h at room temperature in blocking buffer (20 mM Tris-HCl (pH 7.5), 450 mM NaCl, 0.1% Tween-20, and 4% non-fat milk), membranes were incubated overnight at 4°C with mouse monoclonal anti-HA antibodies (dilution 1∶20 [22]), or for 2 h at room temperature with rabbit anti-Ptc antiserum (Ab130) (1∶1000) or rabbit polyclonal anti-Smo antibodies (1∶3000), and washed twice in blocking buffer before incubation for 2 h at 4°C with polyclonal anti-mouse (1∶5000) or polyclonal anti-rabbit (1∶3000) immunoglobulin coupled to horseradish peroxidase (Dako). Detection was with an ECL kit (Millipore) and the Fusion FX7 system® (Vilber-Lourmat).

### Gas chromatography analysis

NIH 3T3 cells were cultured to 80–90% confluence, and 30 nM of pure ShhN protein was added to the cell medium 24 h before harvesting. Total extracts were prepared and the lipids were extracted [26]. Lipids were saponified with 1 M methanolic KOH for 16 h at room temperature under argon in the dark. The unsaponified fraction was extracted with *tert*-butyl methyl ether. An aliquot of this fraction was converted to the trimethylsilyl ether derivative to quantify cholesterol by gas chromatography (GC) using a 5890 Series II Hewlett-Packard GC (Agilent) equipped with a flame ionization detector and a DB5-MS fused silica capillary column (30 m×0.25 mm i.d., 0.25 ìm, J&W Scientific) using 5á-cholestane as the internal standard. The chromatographic data were processed using Galaxie software (Varian).

### BODIPY-cholesterol fluorescence measurements

BODIPY-cholesterol was synthesized as described previously [27] and afterwards was purchased (TopFluor-Cholesterol, Avanti Polar Lipids). Stock solutions were prepared at 5 mM in DMSO.

NIH 3T3 cells were cultured in 24-well plates and loaded with 2.5 µM of BODIPY-cholesterol in culture medium for 2 h at 37°C. Cells were rinsed twice with physiological buffer (140 mM NaCl, 5 mM KCl, 1 mM CaCl_2_, 1 mM MgSO_4_, 5 mM glucose, 20 mM HEPES, pH 7.4), and incubated with the same buffer supplemented or not with 30 nM of ShhN for 1 h at 37°C under shaking at 50 rpm. The cell supernatant was centrifuged for 5 min at 6800 *g*, and the BODIPY fluorescence intensity in the supernatants was measured in a Plate reader (Fluostar, Labtech) (excitation 490±10 nm; emission 520±20 nm). For fluorescence microscopy observations, NIH 3T3 cells were cultured on glass plates, loaded with 2.5 µM BODIPY-cholesterol for 2 h at 37°C, rinsed, and incubated with physiological buffer supplemented or not with 30 nM ShhN at 37°C for 30 min. Cells were rinsed with phosphate buffer (pH 7.4), fixed with 4% paraformaldehyde for 10 min, and observed using a deconvolution microscope (DeltaVision) equipped with an objective 60x/1.4 oil. BODIPY fluorescence was analyzed using Image J software.

*S. cerevisiae* over-expressing human Ptc or mouse Myodulin were grown at 18°C until an OD_600_ of 7, washed with cold water, and re-suspended at an OD_600_ of 10 in Hepes-NaOH buffer (pH 7.0) supplemented or not supplemented with 5 mM of 2-deoxy-D-glucose. Cells were incubated with 5 µM BODIPY-cholesterol or 5 µM BODIPY-cyclopamine (BODIPY-CPN, Toronto Research Chemicals Inc) for 2 h at room temperature on a rotating wheel and washed quickly, re-suspended in the same buffer, and incubated at room temperature on the rotating wheel. Aliquots were centrifuged for 1 min at 18,000 *g* immediately after re-suspension and the pellets were solubilized with SDS to measure the BODIPY-cholesterol loaded into yeast. The rest of the samples were centrifuged for 1 min at 18,000 *g*, 30 s, 3, 5 or 20 min after re-suspension, and the resulting supernatants were collected to measure the BODIPY-cholesterol efflux. The BODIPY fluorescence intensity was measured in the Plate reader (Fluostar, Labtech) (excitation 490±10 nm; emission 520±20 nm). For all BODIPY-cholesterol efflux experiments, the results were analyzed using the Student's *t* test where significance is attained at *p*<0.05.

### Surface plasmon resonance (SPR) experiments

SPR experiments were performed on a Biacore 3000 instrument (Biacore/GE Healthcare Uppsala, Sweden). A stock solution of thiocholesterol (Sigma) in DMSO (1 mg/mL) was prepared and diluted 1∶50 in sodium borate buffer, pH 8.5, for covalent thiol-coupling to the flow cell 2 (fc2) of a CM5 sensor chip. The flow cell 1 (fc1) was activated without thiocholesterol and used as a reference for nonspecific binding. Experiments with purified proteins were performed at 10°C. Purification buffer containing 50 mM Tris-HCl (pH 7.4), 150 mM NaCl, 10% glycerol, and 2 mM of DDM was used as the running buffer. Purified proteins (50 µL) were injected on fc1 and fc2 at a flow rate of 10 µL/min, and the differences between sensorgrams on fc2 and fc1 were recorded. The sensor chip surface was regenerated twice with 20 µL of 50 mM NaOH and 0.05% SDS injected at 20 µL/min. For inhibition assays, purified hPtc was incubated with increasing concentrations of pure ShhN protein before injection onto fc1 and fc2. The inhibition percentage of the binding response was plotted vs. ShhN concentration, and the apparent K_i_ value was determined from non-linear regression curve fitting using Origin software.

## Results

### Cholesterol concentration variations affect Smoothened enrichment in the plasma membrane

**Figure 1:**
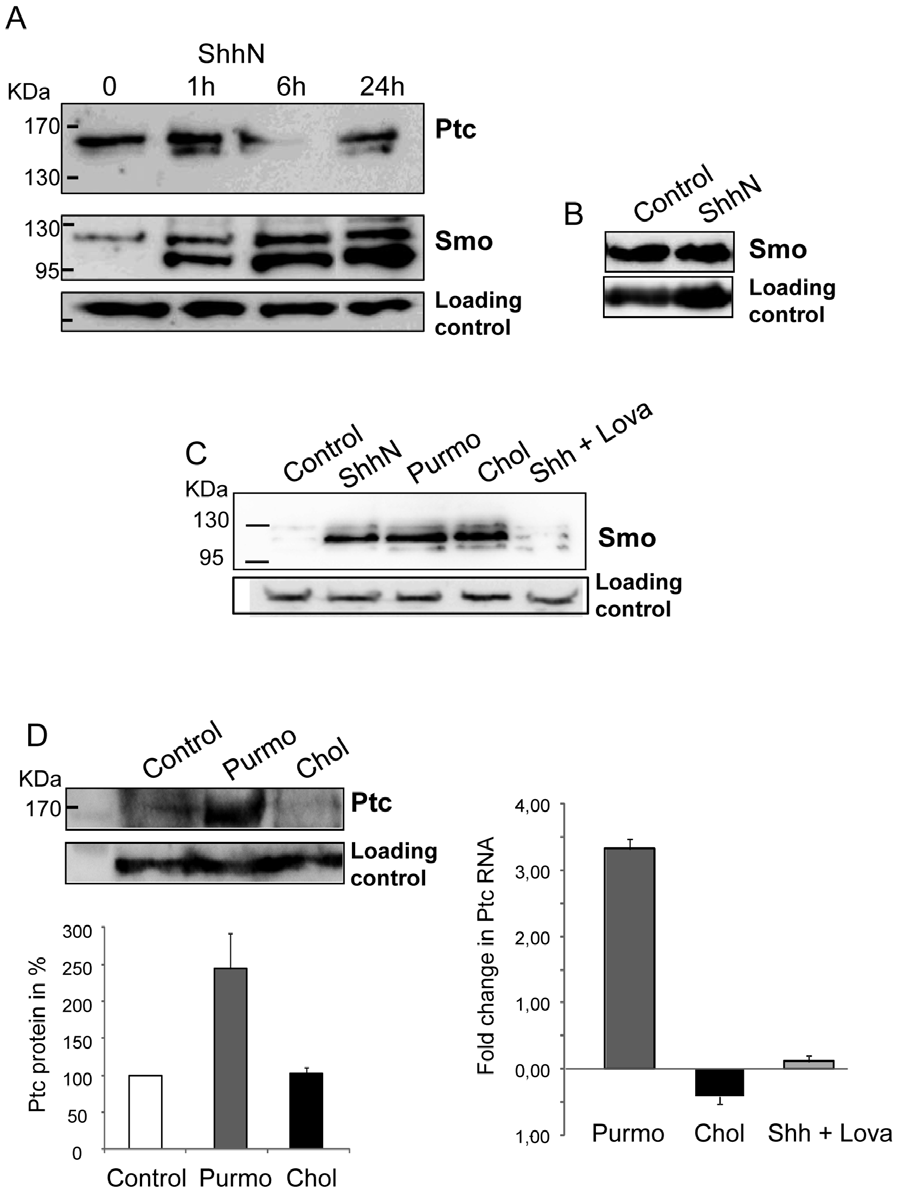
Effect of Hh signaling modulators on Ptc and Smo. ShhN acts on the levels of Ptc and Smo at the plasma membrane (A). Enriched plasma membrane fractions were prepared from NIH 3T3 cells (control and cells treated with 30 nM ShhN for 1, 6, or 24 h) for immunoblotting with antibodies against Ptc or Smo. ShhN does not modify the Smo total amount (B). Total extract from NIH 3T3 cells treated or not treated with 30 nM ShhN for 6 h were prepared for immunoblotting with antibodies against Smo. Cholesterol enhances Smo enrichment at the plasma membrane (C). Enriched plasma membranes fractions were prepared from NIH 3T3 cells (control and cells treated for 6 h with 30 nM ShhN, 6 h with 10 µM purmorphamine (a Smo agonist), 3 h with 2.5 µM cholesterol, or 6 h with 30 nM ShhN plus 10 µg/mL of the cholesterol biosynthesis inhibitor lovastatin) for immunoblotting with antibodies against Smo. Contrary to the Hh signaling activators such as purmorphamine, cholesterol does not enhance Ptc expression (D). NIH 3T3 cells were treated for 48 h with 10 µM purmorphamine, with 2.5 µM cholesterol, or with 30 nM ShhN plus 10 µg/mL of lovastatin. Enriched membrane fractions were prepared for immunoblotting with antibodies against Ptc and the Ptc signal was quantified using Image J software from 3 independent experiments (left panel). RT PCR analysis were performed and quantified using 18S mRNA as an endogenous control (right panel).

To investigate the effect of cholesterol modulation on the Hh signaling, we used mouse fibroblasts NIH 3T3, which do not synthesize Shh but are highly responsive to Shh and have been widely used to study the Hh pathway [1], [13], [28], [29], [30]. We purified the amino-terminal domain of the murine Shh protein without cholesterol and palmitate modification (ShhN), and showed that purified ShhN fully activates the Hh signaling [22]. In the absence of ShhN, Smo is inactivated and cleared from the cell surface via endocytosis [31], [32]. Therefore, Smo is only slightly present in the plasma membrane, as shown in Fig. 1A, lane 1. When added to the culture medium, ShhN binds to Ptc and rapidly induces Ptc internalization and degradation (Fig. 1A, lane 3). After 24 h, a dual effect of ShhN is observed on Ptc protein: enhancement of Ptc expression induced by Hh pathway activation and degradation of Ptc due to the presence of ShhN in the culture medium. This explains why the Ptc signal at the plasma membrane increases in comparison with the Ptc signal after 6 h exposure to ShhN but is still lower than that in non-treated cells (Fig. 1A, lane 4). The clearance of Ptc allows Smo enrichment in the plasma membrane (Fig. 1A, lane 2), without changes in Smo total amount (Fig. 1B) [10], [13], [33], [34], [35]. Smo enrichment in the plasma membrane, and more particularly at the primary cilia in vertebrates, is correlated with Hh signaling activation [10], [12], [29]. We incubated fibroblasts NIH 3T3 with ShhN, cholesterol, the Hh pathway agonist purmorphamine, or with ShhN protein plus lovastatin, an inhibitor of cholesterol biosynthesis [36], and we examined the effects on Smo enrichment in the plasma membrane (Fig. 1C). Remarkably, the amount of endogenous Smo at the cell surface increases after incubation of cells with cholesterol in a comparable way to when cells are incubated with ShhN protein or with the Smo agonist purmorphamine (Fig. 1C, lanes 2, 3, 4). On the other hand, addition of lovastatin in the culture medium strongly inhibits Smo enrichment in the plasma membrane by ShhN (Fig. 1C, lane 5). These results show that increasing intracellular cholesterol allows Smo enrichment in the plasma membrane; in contrast, the decrease in intracellular cholesterol concentration induced by addition of lovastatin to the culture medium containing ShhN prevents Smo enrichment in the plasma membrane. This suggests that the intracellular cholesterol concentration may be critical for Smo enrichment in the plasma membrane. Smo agonists such as purmorphamine turn on the expression of Hh pathway target genes like Ptc (Fig 1D, left panel, lane 2); however, we did not observe a boost of Ptc expression in cells treated with cholesterol (Fig. 1D, left panel, lane 3). RT PCR analysis (Fig. 1D, right panel) shows that cholesterol does not significantly affect Ptc RNA expression; in contrast, purmorphamine enhances it more than 3 fold, in good agreement with the Western blot analysis. It also shows that lovastatin treatment prevents the activation of Ptc RNA expression by ShhN. These results suggest that cholesterol is necessary for Smo enrichment in the plasma membrane but is not sufficient for Hh pathway activation.

### Shh decreases cholesterol efflux from mouse fibroblasts

**Figure 2:**
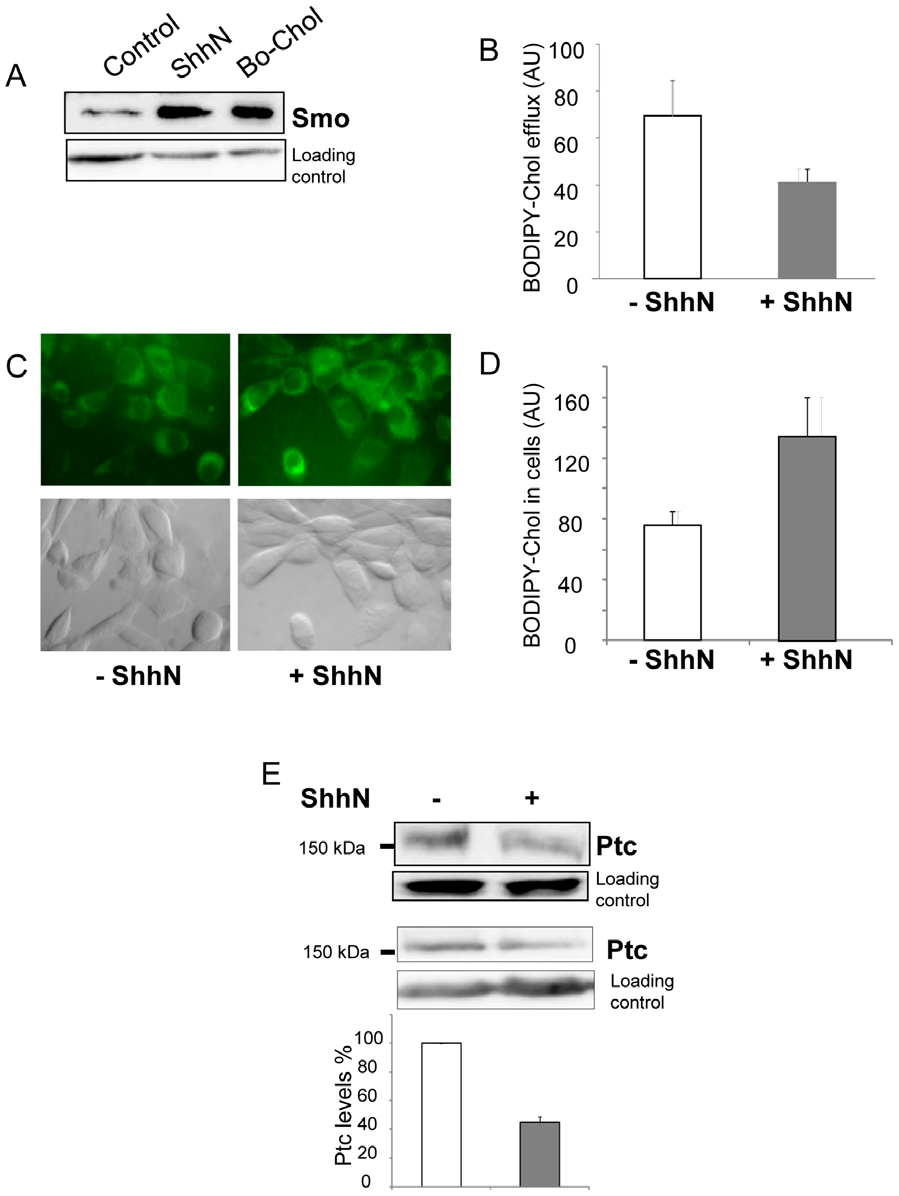
BODIPY-cholesterol efflux from fibroblasts is affected by ShhN. BODIPY-cholesterol induces the accumulation of Smo in the plasma membrane as cholesterol (A). Plasma membrane enriched fractions from control NIH 3T3 (A, lane 1), NIH 3T3 treated for 6 h with 30 nM ShhN (A, lane 2), and NIH 3T3 treated for 3 h with 2.5 µM BODIPY-cholesterol (A, lane 3) were prepared for immunoblotting with antibodies against Smo. ShhN decreases BODIPY-cholesterol efflux (B). NIH 3T3 were cultured in 24-well plates, incubated for 2 h with 2.5 µM BODIPY-cholesterol, and rinsed. Physiological buffer with or without 30 nM ShhN was added to the wells, and the BODIPY fluorescence intensity of the supernatants was measured after 1 h. The mean ± SEM of 8 independent experiments is presented (p = 0.006). The BODIPY fluorescence intensity that remained in NIH 3T3 after 1 h efflux was observed using Delta vision fluorescent microscope (C) and quantified using Image J software (D). The results show that cells treated with ShhN retain more BODIPY-cholesterol than untreated cells. Immunoblotting with antibodies against Ptc of enriched plasma membrane fractions (E, upper panel) and total extracts (E, lower panel) from NIH 3T3 in the presence or absence of 30 nM ShhN show that the level of Ptc protein is decreased by ShhN treatment. The Ptc signal was quantified using Image J software from 3 independent experiments and untreated cells as control.

In order to understand the link between cholesterol and Hh signaling, we used a fluorescent derivative of cholesterol, BODIPY-cholesterol [27]. This cholesterol derivative has been shown to closely mimic the membrane partitioning and trafficking of cholesterol, and, because of its excellent fluorescent properties, to enable direct monitoring of sterol movement by time-lapse imaging [37]. Fibroblasts treated with BODIPY-cholesterol show an enrichment of Smo in the plasma membrane similar to that induced by ShhN treatment (Fig. 2A), indicating that this probe behaves like cholesterol in Hh signaling. NIH 3T3 fibroblasts were grown in 24-well plates, loaded with BODIPY-cholesterol, rinsed, and incubated with physiological buffers with and without ShhN protein. The BODIPY fluorescence measured in the supernatants after 1 h was significantly lower in wells where ShhN protein was added compared to wells that did not contain ShhN (mean decrease 45±6%) (Fig. 2B). The observation of cells by fluorescence microscopy shows that the content of BODIPY-cholesterol is significantly higher in cells incubated with a buffer containing ShhN protein (Fig. 2 C, D). These results are in good agreement with the data obtained by gas chromatography which show that cells treated with ShhN contain a higher intracellular cholesterol concentration compared with untreated cells (Table 1), and suggest that ShhN treatment reduces cholesterol efflux from cells. ShhN protein has been shown to bind to Ptc, and to induce its internalization and degradation [13], [38], [39]. Despite the weakness of the signals due to the low basal expression of Ptc, we observed that ShhN treatment indeed decreased the amount of Ptc both in membrane preparations (Fig. 2 E, upper panel) and total extracts (Fig. 2 E, lower panel) to 44±4% of Ptc signal obtained with untreated fibroblasts.

**Table 1:** Shh increases cell cholesterol concentration.

Experiment	Cholesterol (µg/µg prot)		Variation (%)
	*Control*	*+ Shh*	*Shh/control*
1	**0.0061**	**0.0083**	**+36.13**
2	**0.0157**	**0.0177**	**+12.58**
3	**0.0156**	**0.0190**	**+21.57**

NIH 3T3 cells were treated with or without 30 nM ShhN and the cholesterol in cell extracts was quantified by gas chromatography in three independent experiments.

### Treatment of fibroblasts with Hh pathway modulators modify cholesterol efflux

**Figure 3:**
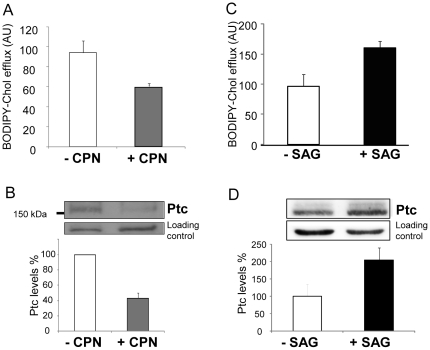
Fibroblast treatment with Hh pathway modulators modifies BODIPY-cholesterol efflux. NIH 3T3 fibroblasts were cultured in 24-well plates for BODIPY-cholesterol efflux measurements (A and C) or in 100-mm diameter plates for membrane preparations and immunoblotting (B and D), and treated for 48 h with 10 µM cyclopamine (CPN) (A and B) or 100 nM SAG (C and D). CPN and SAG are antagonists and agonists of the Hh pathway, respectively. The histograms presented in A and C are the mean ± SEM of 3 independent experiments (p = 0.04). The Ptc signals from immunoblots presented in B and D and other independent experiments were quantified using Image J software and untreated cells as control.

NIH 3T3 fibroblasts were treated for 48 h with cyclopamine (CPN, a well-known antagonist of the Hh pathway [40]) or SAG (a well-known Hh pathway agonist [19]), before incubation with BODIPY-cholesterol. After rinsing, the cells were incubated for 1 h in physiological buffer. We observed that the BODIPY fluorescence in the supernatants was significantly lower in wells containing cells treated with CPN compared to wells containing untreated cells (mean fluorescence decrease of 35±6%) (Fig. 3A), and significantly higher in wells containing cells treated with SAG compared to wells containing untreated cells (mean fluorescence increase of 68%) (Fig. 3C). These results indicate that treatment of cells with the antagonist CPN decreases BODIPY-cholesterol efflux, whereas treatment with the agonist SAG enhances BODIPY-cholesterol efflux. We also observed that Ptc expression level was reduced to 45±12% in NIH 3T3 cells treated with CPN (Fig. 3B) and enhanced to 205±35% in NIH 3T3 cells treated with SAG (Fig. 3D) in comparison with Ptc signal obtained with untreated cells. The binding of CPN to Smo inhibits Hh signal transduction and represses the expression of Hh target genes such as Ptc. On the other hand, the binding of SAG to Smo activates Hh signal transduction and the expression of Ptc.

The results obtained with NIH 3T3 fibroblasts show that modulating the Hh signaling and Ptc protein levels has an effect on intracellular cholesterol content and BODIPY-cholesterol efflux. To demonstrate the link between Ptc and cholesterol efflux, we over-expressed human Ptc in yeast and analyzed the effect of Ptc expression on the efflux of cholesterol from yeast cells.

### Human Ptc expressed in yeast increases the efflux of cholesterol

**Figure 4:**
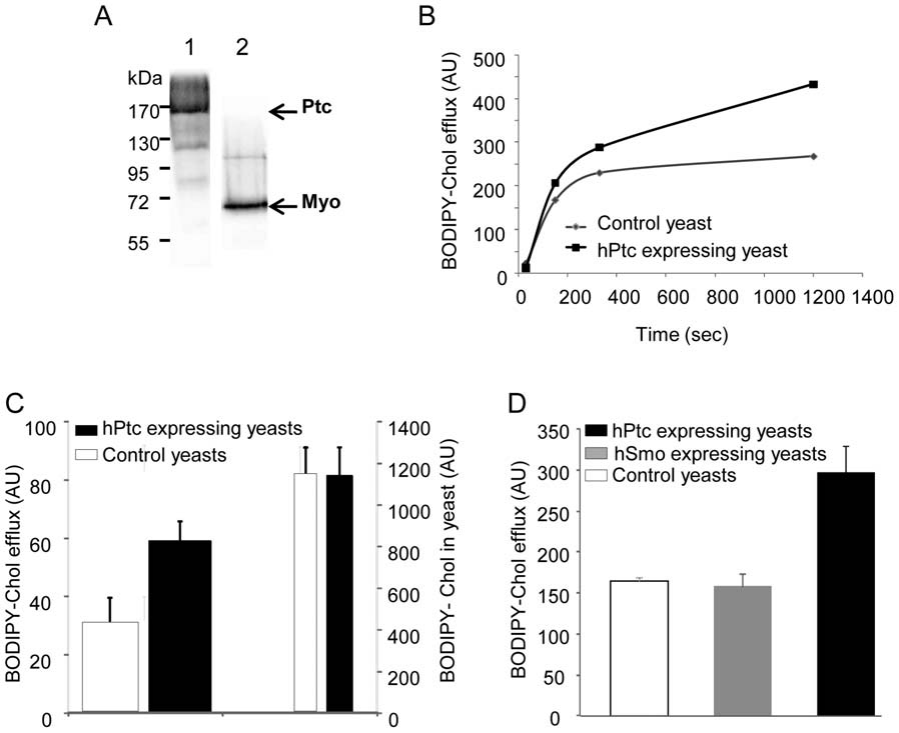
hPtc over-expressed in *S. cerevisiae* induces cholesterol efflux. Membranes from human Ptc (hPtc) expressing yeast (A, lane 1) and mouse myodulin (Myo) expressing yeast (used as control) (A, lane 2) were prepared for immunoblotting with antibodies against hemagglutinin (A). Yeast over-expressing Myo (control) or hPtc were incubated for 2 h with 2.5 µM BODIPY-cholesterol, washed, re-suspended in buffer, and then centrifuged for 30 s and for 3, 5, or 20 min. Then, the fluorescence intensity of the supernatants was measured (B). The BODIPY fluorescence intensity of the supernatants 20 min after resuspension and in yeast after loading and washing was measured in 9 independent experiments. The mean values ± SEM are presented (p = 0.002) (C). The same experiments were carried out with yeast expressing human Smo and in the presence of 5 mM 2-deoxy-D-glucose to inhibit ABC transporters during BODIPY-cholesterol loading and efflux (D).

We recently developed the functional expression of the human protein Ptc (hPtc) in the plasma membrane of *S. cerevisiae* with a multitag affinity purification sequence (MAP) fused in its C-terminus (Fig. 4A, lane 1). We previously showed that hPtc was able to bind its ligand ShhN both in yeast membranes and in surfactant suspension after purification [22], [41]. Yeast expressing the non-relevant membrane protein Myodulin (Myo) [42] (used as control) and yeast expressing hPtc were loaded with BODIPY-cholesterol, rinsed, re-suspended with buffer, and centrifuged for 30 s, and for 3, 5, and 20 min. We observed that the BODIPY fluorescence intensity in the supernatants of yeast expressing hPtc was higher than that of yeast expressing Myo (Fig. 4B). We then measured the BODIPY-cholesterol fluorescence intensity present in the supernatants 20 min after re-suspension in nine independent experiments. We observed that the presence of hPtc significantly increased BODIPY-cholesterol efflux (about 88%), while the content of BODIPY-cholesterol incorporated in yeast expressing Myo or hPtc were comparable (Fig. 4C). We then measured BODIPY-cholesterol efflux in the presence of 2-deoxy-D-glucose in order to inhibit ABC transporters, and we obtained comparable results (Fig. 4D), suggesting that the BODIPY-cholesterol efflux observed in yeast expressing hPtc is not linked to ABC transporter activity. We also carried out BODIPY-cholesterol efflux measurements on yeast cells over-expressing human Smo (hSmo) [24]. We observed that the expression of hSmo has no effect on BODIPY-cholesterol efflux, which is comparable to that measured in yeast expressing Myo and lower than that measured in hPtc-expressing yeast (Fig. 4D). Similar experiments were carried out with another BODIPY-modified cholesterol related molecule (BODIPY-cyclopamine), and on yeast cells treated with cyclopamine before incubation with BODIPY-cholesterol. The BODIPY fluorescence intensity measured in the supernatants 20 min after incubation with BODIPY-cyclopamine and re-suspension was not significantly different with yeast expressing hPtc than with yeast expressing Myo (Fig. S2). CPN treatment did not prevent BODIPY-cholesterol efflux enhancement observed in yeast expressing Ptc (Fig. S3). These observations indicate that CPN does not have a direct effect on BODIPY-cholesterol efflux from fibroblasts. Furthermore, these results strongly suggest that human Ptc contributes to BODIPY-cholesterol efflux when over-expressed in yeast.

### Ptc binds to cholesterol

**Figure 5:**
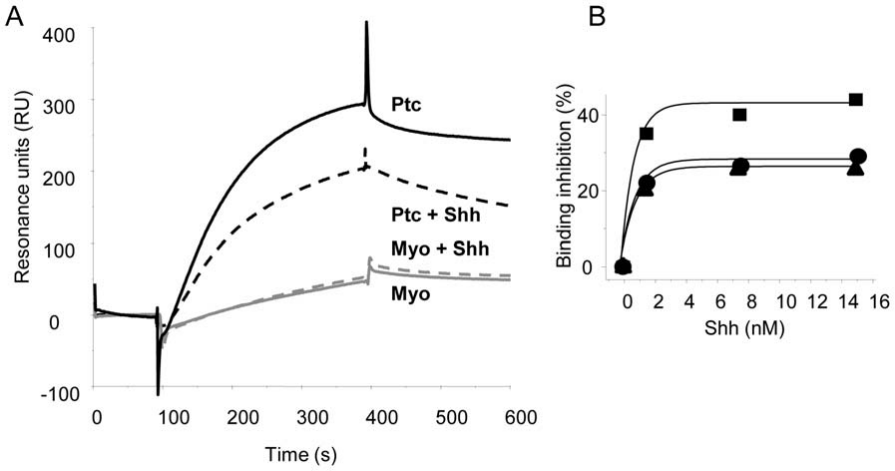
Purified Ptc binds to cholesterol. Human Ptc and mouse Myodulin were purified from *S. cerevisiae*. Proteins were injected onto the flow cell 2 (fc2) covalently coupled with thiocholesterol and the flow cell 1 (fc1) used as control of a Biacore sensor chip CM5. The difference between the sensorgrams recorded on fc2 and fc1 are reported (A). The sensorgrams recorded with Ptc (black lines) and Myodulin (grey lines) before (full lines) and after (tear lines) incubation with 15 nM pure ShhN show that Ptc binds to cholesterol coupled onto the sensor chip and that ShhN partially inhibits this binding. Sensorgrams were recorded with Ptc incubated with increasing concentrations of pure ShhN (1.5, 7.5, 15 nM) before injection, and the% cholesterol binding inhibition was plotted vs. ShhN concentration. The curves obtained from 3 independent experiments were fitted on an exponential curve using Origin software, giving K_i_ values between 0.8 and 1.0 nM (B).

We then examined the ability of hPtc to bind to cholesterol using Surface Plasmon Resonance (SPR). Myo and hPtc were purified from yeast, and injected onto a Biacore sensor chip in which one flow cell (fc) was covalently coupled to thiocholesterol and another was just activated without thiocholesterol for non-specific binding. The sensorgrams resulting from the difference between the binding on the thiocholesterol-coupled fc and the control one showed a clear binding of hPtc to cholesterol coupled on the fc in comparison with Myo (Fig. 5A). Incubation of hPtc with pure ShhN protein prior to injection onto the sensor chip inhibited the binding of hPtc to cholesterol in a ShhN concentration-dependent manner, while ShhN had no effect when incubated with Myo prior to injection (Fig. 5A). The apparent inhibition constants (K_i_) calculated for three independent experiments were between 0.8 and 1 nM (Fig. 5B), which corresponds to the apparent affinity of ShhN for hPtc [15], [41]. These results suggest that hPtc binds to cholesterol, and that the interaction of ShhN with hPtc partially prevents this binding, probably by inducing a conformational change in Ptc before its internalization and degradation in cells.

## Discussion

Since the first publication on cholesterol balance in mice [43], it is clear that cholesterol homeostasis is one of the most intensely regulated processes in biology [44]. Perturbations of cholesterol biosynthesis induce severe developmental malformations in a multitude of diseases such as Smith-Lemli-Opitz syndrome (SLOS), desmosterolosis, and lathosterolosis. SLOS and lathosterolosis are characterized by accumulation of the cholesterol precursors 7-dehydrocholesterol and lathosterol, and decreased cholesterol concentration, which also impairs the Shh pathway at the level of Smo [21]. Holoprosencephaly (HPE), the most severe form of SLOS, results from impairment of Shh signaling secondary to abnormal cholesterol metabolism [45], [46]. These observations and studies reporting the effect of cholesterol or oxysterols on the Hh pathway [18] indicate that cholesterol or its derivatives are important components for the function of the Shh signaling cascade. The question is: how does cholesterol intervene in the Hh pathway?

We report here that activation of Hh signaling with the purified Shh N-terminal active domain increases the endogenous intracellular concentration of cholesterol in mouse NIH 3T3 fibroblasts, and that adding cholesterol to the culture medium induces Smo enrichment in the plasma membrane, as does ShhN or the Smo agonist purmorphamine. In contrast, treatment of cells with an inhibitor of the cholesterol synthesis (lovastatin) prevents Smo accumulation in the plasma membrane by ShhN. These results suggest that Smo enrichment in the plasma membrane, which is a critical step in Hh signal transduction [10], [13], [33], [34], depends on the intracellular concentration of cholesterol. This hypothesis is in good agreement with studies reporting activation of the Hh pathway by oxysterols [13], [18], [19] and inhibition of the cellular response to Hh protein stimulation by genetic or pharmacological sterol depletion [21], [45], [47]. However, we did not observe an increase of Ptc protein or RNA expression after cholesterol treatment, contrary to purmorphamine treatment (which enhanced Ptc RNA and protein expression by approximately 3 fold). This was expected for an agonist of Hh signaling since the *Ptc* gene is one of the Hh target genes. Moreover, we observed that decreasing cholesterol concentration by lovastatin treatment prevents the activation of Ptc expression by ShhN, which is surely a consequence of inhibition of Smo accumulation at the plasma membrane. These results suggest that intracellular cholesterol, by promoting Smo enrichment in the plasma membrane, is necessary for Hh signal transduction but is not sufficient for Hh pathway activation. Our results are in full agreement with those of Rohatgi and co-workers, who reported that a second step is required after Smo enrichment in cilia for full Hh pathway activation [29], and with those of Corcoran and Scott, who reported that cholesterol or specific oxysterols are required for Shh signal transduction in medulloblastoma cells and that these sterols would act at the level of Smo [18].

In the second part of this report, we sought to demonstrate that Ptc could be responsible for the reduction of the cholesterol concentration in the environment of Smo by pumping cholesterol out of the cell. Therefore, we used BODIPY-cholesterol, a fluorescent cholesterol derivative in which the BODIPY moiety is linked to C-24 of the cholesterol side chain [27]. This probe was found to mimic endogenous cholesterol partitioning into liquid-ordered domains in model membranes and allowed visualization of trafficking of cholesterol in living cells [37]. We observed that the presence of ShhN causes an inhibition of BODIPY-cholesterol efflux from NIH 3T3 fibroblasts. We also noted a decrease of the amount of Ptc protein in these cells, which is in good agreement with studies reporting that ShhN induces Ptc internalization and degradation [13], [33], [34], [48]. Furthermore, treatment of NIH 3T3 fibroblasts with the Smo antagonist cyclopamine inhibits Ptc expression, which was expected since the *Ptc* gene is one of the targets of the Hh pathway, and reduces BODIPY-cholesterol efflux. Therefore, we thought that the decrease of BODIPY-cholesterol efflux observed with fibroblasts treated with ShhN or with cyclopamine could be due to the clearance of Ptc from the plasma membrane. Remarkably, treatment of fibroblasts with the Hh pathway agonist SAG, which enhances Ptc protein expression, clearly increases BODIPY-cholesterol efflux, and over-expression of human Ptc in yeast also significantly enhances BODIPY-cholesterol efflux from yeast. Finally, we showed that Ptc is able to bind cholesterol using purified hPtc and surface plasmon resonance measurements. All of these results strongly suggest that Ptc may be involved in cholesterol efflux from cells.

Moreover, the sequence similarities of Ptc with the Niemann-Pick disease type C1 protein NPC1, which is involved in binding and transport of cholesterol [49], [50], [51], [52], support our hypothesis of the involvement of Ptc in cholesterol binding and transport. The Ptc sequence possesses a sterol-sensing domain (SSD) which is a phylogenetically conserved domain shared by several classes of proteins having key roles in different aspects of cholesterol homeostasis, such as NPC1 [53], and is essential for Smo repression in *Drosophila* and vertebrates [54], [55]. Indeed, blocking Ptc SSD activity in *Drosophila* not only causes endosomal lipid accumulation but also alters the trafficking of Smo from endosomes [56].

**Figure 6:**
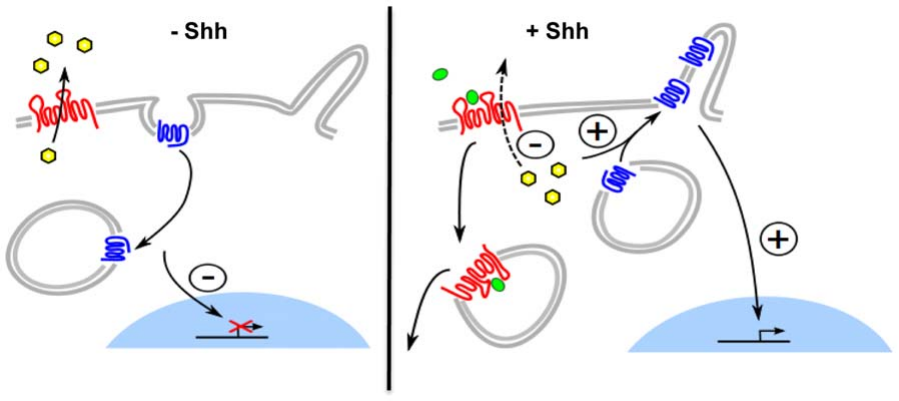
Schematic representation of Ptc/Smo regulation mechanism. Left panel: In the absence of Shh, Ptc (red) is present at the plasma membrane, where it contributes to cholesterol (yellow) efflux from the cell. Smo (blue) is inactivated and traffics between endosomes and the plasma membrane. Right panel: Shh (green) binding to Ptc induces Ptc internalization and inhibits cholesterol efflux. The concentration of cholesterol increases, allowing accumulation of Smo at the cilium membrane.

From the results reported here, we propose the following regulation mechanism between Ptc and Smo (Fig. 6). In the absence of ShhN, Ptc is present in the plasma membrane at or near the primary cilium where it contributes to cholesterol efflux from the cell. This decreases intracellular cholesterol concentration and inhibits Smo, which is cleared from the cell surface via endocytosis [31], [32]. Interaction of ShhN with Ptc induces Ptc internalization and inhibits cholesterol efflux. The intracellular concentration of cholesterol increases, allowing the enrichment of Smo at the cilium.

The involvement of Ptc in cholesterol transport and the Ptc-dependent Smo regulation mechanism proposed here are consistent with the Hh signaling impairment caused by decreased intracellular cholesterol levels observed in congenital malformations such as SLOS and lathosterolosis [21]. Defective regulation of cholesterol biosynthesis could further aggravate impaired Hh signaling in holoprosencephaly [46]. It is also consistent with recent data suggesting that binding of Hh to Ptc de-repressed the levels of phosphatidylinositol 4-phosphate (PI-4P) which in turn promoted Smo activation [20], since cholesterol has been shown to modulate PI-4P synthesis [57].

Finally, several studies have reported that addition of cholesterol biosynthesis inhibitors such as lovastatin to classical chemotherapy enhances the efficacy of treatment for some tumors [58]. Interestingly, we noted that statin-sensitive tumors present an aberrant Hh pathway activation. Our results could explain how the decreased cholesterol concentration induced by statins may affect Smo enrichment at the cell surface, and subsequently inhibit the Hh pathway and the proliferation of cancer cells. Thus targeting the Hh pathway in combination with classical chemotherapy may be a promising therapeutic strategy for treatment of Hh related tumors.

## Supporting Information

Figure S1 **Ab130 antiserum specificity.** Total extracts from HEK293 cells transiently transfected with mouse Ptc cDNA (1) or empty vector (2) were western blotted with antiserum Ab130. The bands that appear in lane 1 correspond to monomeric and multimeric forms of Ptc. These bands are not present when western blotting was carried out using Ab130 serum incubated with the polypeptide used for immunization (lane 3), showing that Ab130 antiserum is a specific anti-Ptc antibody.
(TIF)

Figure S2 **Ptc specifically enhances cholesterol efflux.** Yeast expressing the non-relevant membrane protein Myo (used as control) and yeast expressing human Ptc were incubated with 2.5 µM BODIPY-cholesterol or 2.5 µM BODIPY-cyclopamine (BODIPY-CPN) for 2 h, and the BODIPY fluorescence intensity was measured in the supernatants 20 min after washing and resuspension. The presence of hPtc increased BODIPY-cholesterol efflux by 80% vs. an increase of BODIPY-CPN efflux by only 20%.
(TIF)

Figure S3 **Cyclopamine has no effect on BODIPY-cholesterol efflux in yeast.** Yeast expressing human Ptc were treated with 10 µM cyclopamine before incubation with 2.5 µM BODIPY-cholesterol for 2 h. The BODIPY fluorescence intensity was measured in the supernatants 20 min after washing and resuspension. Cyclopamine treatment did not affect BODIPY-cholesterol efflux.
(TIF)
