# Supplementary figures and images for: The Hedgehog Receptor Patched Is Involved in Cholesterol Transport
Source: PLoS One. 2011 Sep 8;6(9):e23834. doi: 10.1371/journal.pone.0023834 (PMC3169562; doi:10.1371/journal.pone.0023834)

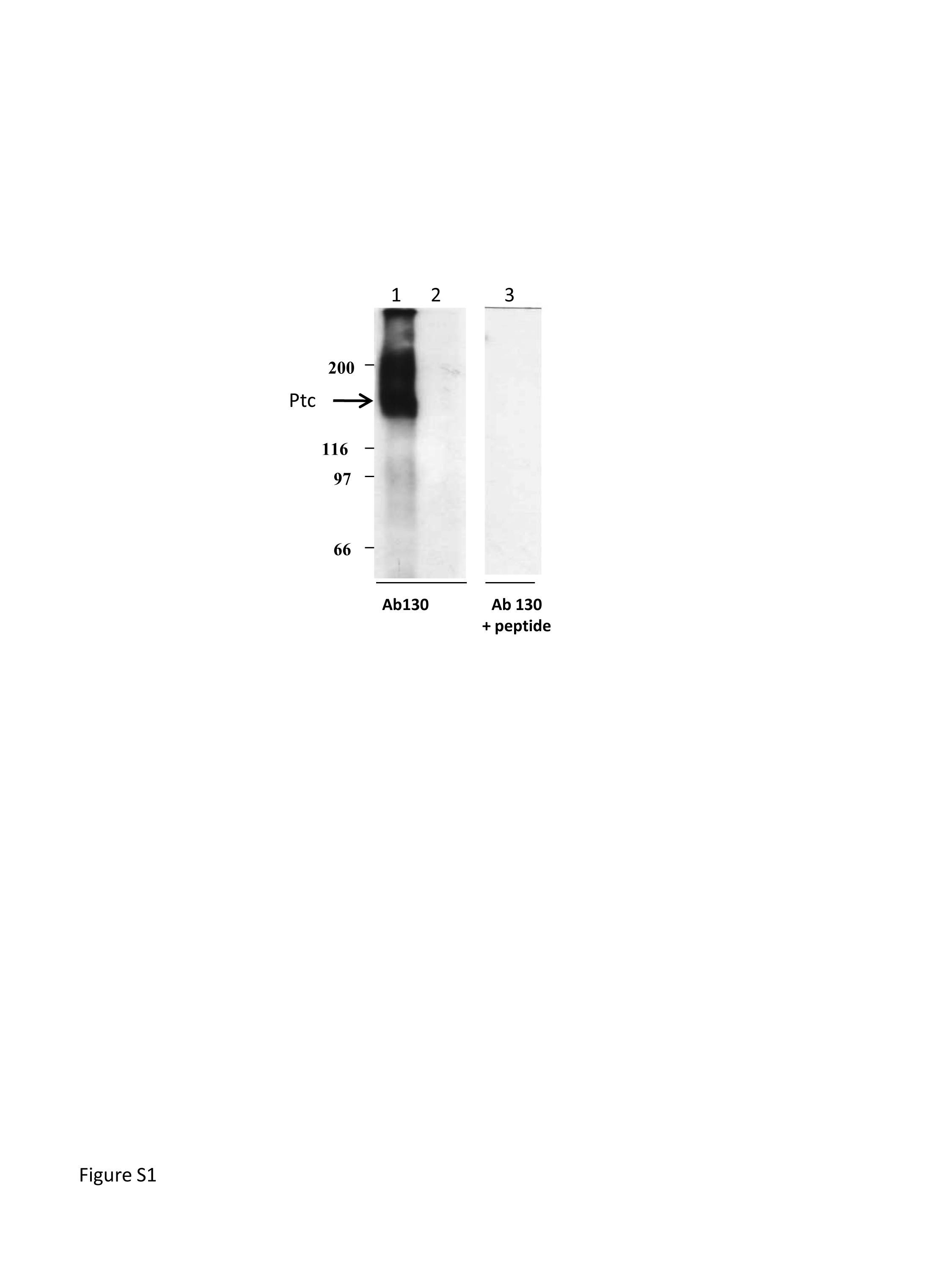

Supplement: Figure S1 — Ab130 antiserum specificity. Total extracts from HEK293 cells transiently transfected with mouse Ptc cDNA (1) or empty vector (2) were western blotted with antiserum Ab130. The bands that appear in lane 1 correspond to monomeric and multimeric forms of Ptc. These bands are not present when western blotting was carried out using Ab130 serum incubated with the polypeptide used for immunization (lane 3), showing that Ab130 antiserum is a specific anti-Ptc antibody. (TIF) [file pone.0023834.s001.tif]

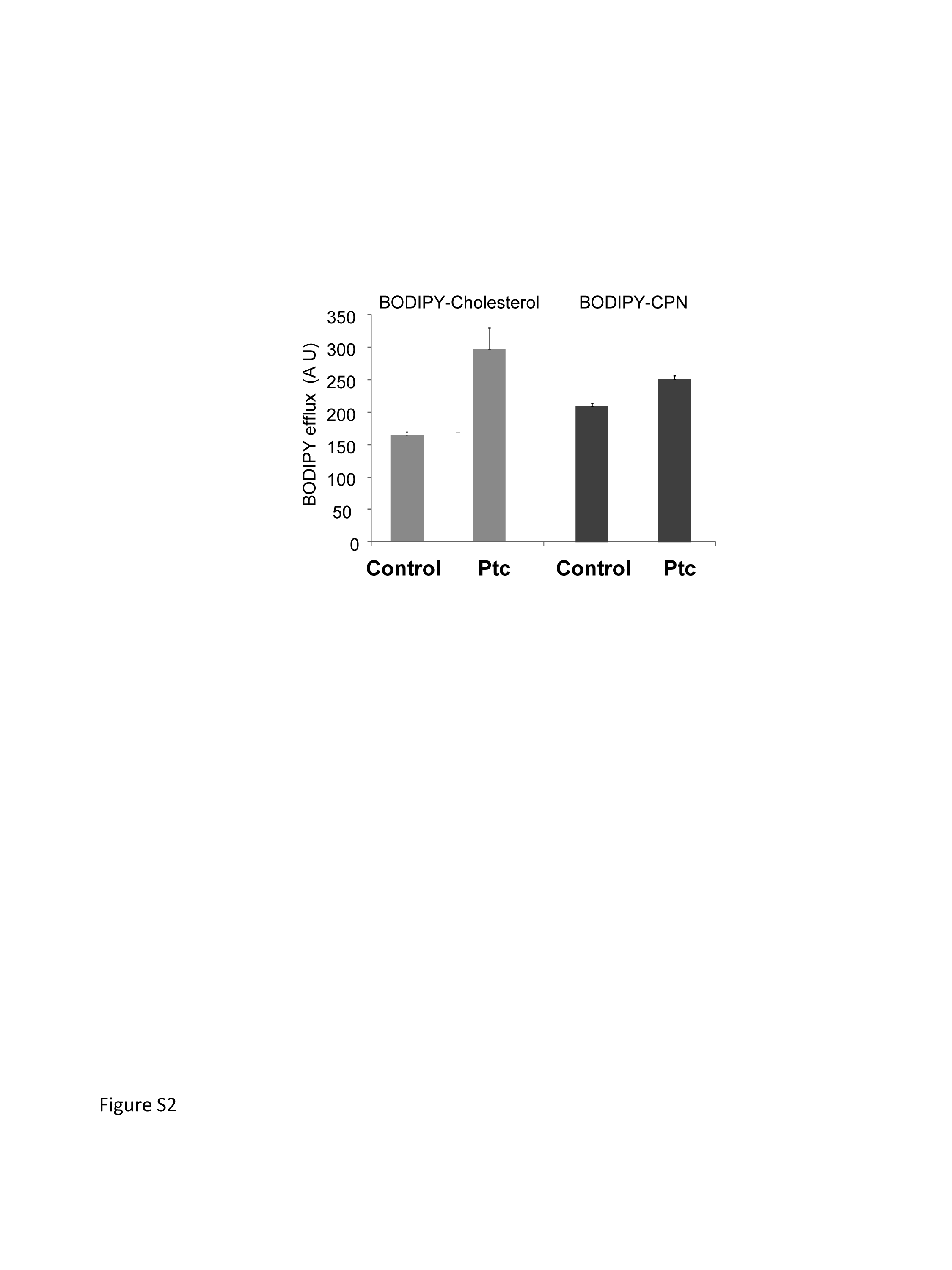

Supplement: Figure S2 — Ptc specifically enhances cholesterol efflux. Yeast expressing the non-relevant membrane protein Myo (used as control) and yeast expressing human Ptc were incubated with 2.5 µM BODIPY-cholesterol or 2.5 µM BODIPY-cyclopamine (BODIPY-CPN) for 2 h, and the BODIPY fluorescence intensity was measured in the supernatants 20 min after washing and resuspension. The presence of hPtc increased BODIPY-cholesterol efflux by 80% vs. an increase of BODIPY-CPN efflux by only 20%. (TIF) [file pone.0023834.s002.tif]

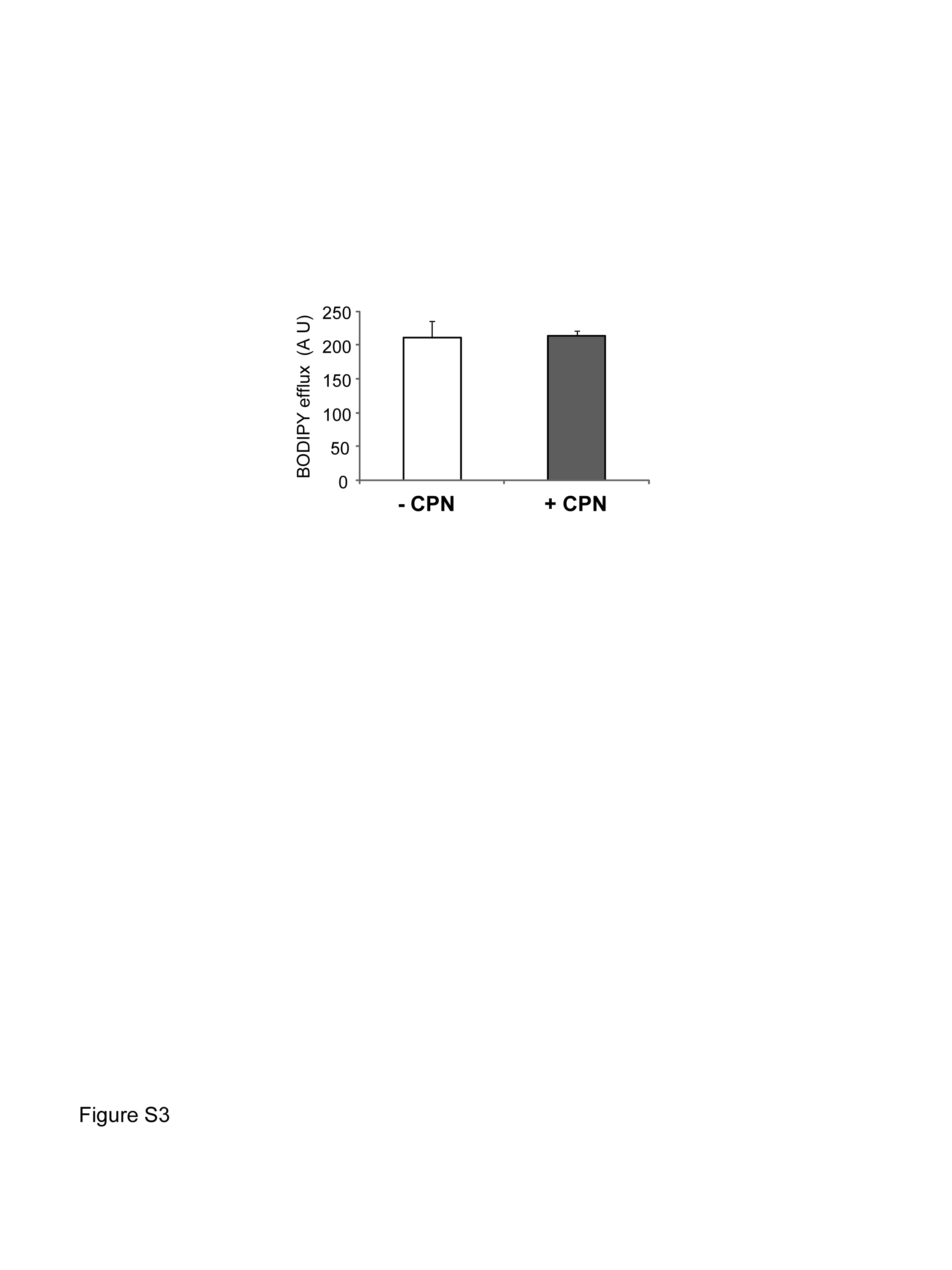

Supplement: Figure S3 — Cyclopamine has no effect on BODIPY-cholesterol efflux in yeast. Yeast expressing human Ptc were treated with 10 µM cyclopamine before incubation with 2.5 µM BODIPY-cholesterol for 2 h. The BODIPY fluorescence intensity was measured in the supernatants 20 min after washing and resuspension. Cyclopamine treatment did not affect BODIPY-cholesterol efflux. (TIF) [file pone.0023834.s003.tif]
